# Cocaine in Cervical Dilatations for Dysmenorrhœa

**Published:** 1888-03

**Authors:** M. J. Birdsong

**Affiliations:** Greenville, Texas


### COCAINE IN CERVICAL DILATATION FOR DYSMENORRHŒA.

By *M. J. Birdsong, M. D., Greenville, Texas.*

For Daniel's Texas Medical Journal.

MRS. G. M. has been the wife of a prominent lawyer of our city for over a year. She is now 20 years old. Menstruated first at 13 years, which has always been painful. On examination I found a long slender cervix with pinhole os; nothing else abnormal obvious.

She was opposed to taking chloroform, so I proposed to use cocaine as a substitute, to which she consented. I made a solution as follows:

℞.—Mur. cocaine grs. x.
Water m. x.
Liquid vaseline ʒ. iv.
M.

I then wrapped two slender whale-bone probangs with absorbant cotton.

I then placed her in Sims' left lateral position with Sims' speculum in place etc. Her husband being the only assistant. I then saturated the cotton on the probangs with the solution above. One of them was introduced fully into the uterus and left in situ. Absorbent cotton was saturated with the solution and packed around the vaginal portion of the cervix and let remain for fifteen minutes, when it was all removed and another application, as above, was used, and allowed to remain the same time, when it was removed also. The dilator was now introduced and dilation began at once, and in a few seconds it was over. But for fear I had not fully dilated the internal os, I closed the instrument and pressed it in farther, and dilated it over. All of which was done with but little pain to patient. In about ten days she menstruated with but little pain, not more than is common for ladies, and is now pregnant and in fine health.

*Second Case*, Jan. 20, 1888 :

Mrs. A. S., a young married lady, complained as in case No. 1. and the technique, the same as in No. 1, I will not repeat. But state that I could not procure the vaseline, and substituted lanoline, which was just as efficient, except it had to be warmed to make it sufficiently soft.

Case No. 2 has not had time to know whether it is a success or not; but as I claim nothing for the operation, it suffices to show the effect of the cocaine.

